# PREDICTING MILD COGNITIVE IMPAIRMENT AND DEMENTIA: MACHINE LEARNING VERSUS TRADITIONAL METHODOLOGIES

**DOI:** 10.1093/geroni/igae098.3323

**Published:** 2024-12-31

**Authors:** Bada Kang, Dahye Hong, Jennifer Kim, Sarah Oh

**Affiliations:** Yonsei University, Seoul, Republic of Korea; Yonsei University, Seoul, Republic of Korea; Yonsei University, Seoul, Republic of Korea; Harvard T.H. Chan School of Public Health, Boston, Massachusetts, United States

## Abstract

Although mild cognitive impairment (MCI) faces significant challenges in terms of early diagnosis and intervention, machine learning (ML) shows promise in managing complex data for MCI and dementia prediction. This study assessed and compared the predictive accuracy of ML models with traditional logistic regression in identifying MCI and dementia onset using the Korean Longitudinal Study of Aging (KLoSA) dataset. The sample comprised 4,975 older adults aged 60 years or older. Identification of MCI and dementia relied on self-reported diagnoses with sociodemographic, lifestyle, and health-related variables as key covariates. The dataset was categorized into training and test sets to predict MCI and dementia, employing models such as logistic regression, light gradient-boosting machine, XGBoost, CatBoost, and Random Forest. Shapley additive explanation values were used to determine the contribution of each feature to the prediction rate. Logistic regression excelled in predicting dementia onset, whereas XGBoost performed better in predicting MCI (XGBoost AUC: 0.7271; Random Forest AUC: 0.6741). Increased assets reduced MCI risk, however, lower education, reduced activities of daily living functional ability, and pain in daily life increased MCI risk. Predictors of developing dementia were being female, older age, reduced activities of daily living functional ability, and lower education. The ML algorithms, particularly XGBoost, demonstrated potential for predicting MCI onset using KLoSA data. This study underscores the crucial role of sociodemographic factors in the early prediction and prevention of cognitive impairment. By leveraging readily available sociodemographic and health-related characteristics, more cost-effective and targeted interventions can be provided to at-risk community-dwelling older adults.

